# Human chorionic gonadotrophin indirectly activates peripheral γδT cells to produce interleukin‐10 during early pregnancy

**DOI:** 10.1002/iid3.1119

**Published:** 2024-01-10

**Authors:** Liman Li, Yuan Liu, Wenjie Zhou, Chuan Yang, Ting Feng, Hong Li

**Affiliations:** ^1^ Center of Translational Medicine, Key Laboratory of Birth Defects and Related Diseases of Women and Children of Ministry of Education, West China Second University Hospital Sichuan University Chengdu China; ^2^ Department of Laboratory Medicine, West China Second University Hospital Sichuan University Chengdu Sichuan China; ^3^ Laboratory of Pulmonary Immunology and Inflammation, Frontiers Science Center for Disease‐Related Molecular Network Sichuan University Chengdu China

**Keywords:** hCG, peripheral γδT cells, CD69, IL‐10, early pregnancy

## Abstract

**Backgrounds:**

The immunomodulatory properties of human chorionic gonadotrophin (hCG) have been identified to be critical for successful pregnancy. However, the effects of hCG on peripheral γδT cells during early pregnancy have not been reported previously.

**Methods:**

We cocultured the purified γδT cells and peripheral blood mononuclear cells (PBMCs) with early pregnancy‐relevant hCG concentrations and investigated the changes in the immune functional characteristics of γδT cells via flow cytometry assays.

**Results:**

The ratios of CD69^+^ and IL‐10^+^ γδT cells were increased in early pregnant women compared to nonpregnant women. γδT cells expressed low levels of the mannose receptor (CD206) instead of the classical hCG/LH receptor for hCG. The direct treatment of purified γδT cells with early pregnancy‐relevant hCG concentrations may have no significant effects on their immune functions. Interestingly, when PBMCs were treated with the same broad range of hCG concentrations, the ratios of CD69^+^ and IL‐10^+^ γδT cells to total γδT cells were significantly increased.

**Conclusion:**

Certain early pregnancy‐relevant hCG concentrations could enhance the ratios of peripheral CD69^+^ and IL‐10^+^ γδT cells, contributing to the activation of γδT cells and immunological tolerance during early pregnancy. However, these affects may not be strongly mediated by direct ligand–receptor interactions and they may highly depend on immune microenvironment. Our novel observations propose a perspective into the endocrine‐immune dialog that exists between the fetus and maternal immune cells.

## Graphical abstract

Peripheral γδT cells contribute to establishing immune tolerance in early pregnancy. Increased hCG concentrations in the blood circulation may not directly affected γδT cells functions because of the low expression of mannose receptor in γδT cells. However, when PBMCs incubated with hCG, certain pregnancy‐relevant hCG concentrations can increase the ratios of Ki67+γδT cells, CD69^+^γδT cells, and IL‐10^+^γδT cells, which might be dependent on the immune microenvironment modulation.

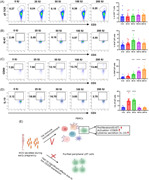

## 1 INTRODUCTION

The presence of an allogeneic fetus poses a significant challenge to the immune system of the mother.^1^ Various maternal immune cells, including natural killer (NK) cells, macrophages, T cells, and dendritic cells, play crucial roles in establishing immune tolerance during the early stage of pregnancy.^2^ Among these immune cell subsets, γδT cells are particularly abundant in decidua, with proportions reaching up to 60% in CD3^+^T cells.^3^, ^4^ γδT cells demonstrate a diverse array of immunomodulatory capabilities in pregnancy.^5^ For instance, they possess the ability to secrete various anti‐inflammatory factors, including interleukin‐10 (IL‐10) and transforming growth factor‐β (TGF‐β), in both human and mouse models, thereby promoting the Th2 response.^6^, ^7^ In addition, the interactions between the receptors on γδΤ cells and the nonclassical HLA molecules on trophoblast cells play crucial roles in facilitating maternal tolerance induction.^8^ γδΤ cells also exhibit cytotoxic potency and contribute to the anti‐infection defense during pregnancy.^9^ Consequently, dysfunctional γδT cells are increasingly recognized as significant contributors to the development of pregnancy‐related complications.^10^, ^11^, ^12^ A promising avenue of research involves the identification of factors that regulate the immune functions of γδT cells in early pregnancy.

Human chorionic gonadotrophin (hCG), one of the earliest proteins secreted by fetal trophoblasts, is a heterodimeric glycoprotein with a unique beta subunit and an alpha subunit.^13^, ^14^ During normal pregnancy, hCG is detectable in maternal serum after the initiation of embryo implantation. Then, hCG rapidly rises to a peak value of approximately 8–10 weeks gestation and gradually decreases in the second trimester.^15^ The primary role of hCG is to stimulate the corpus luteum in the ovary to produce progesterone.^16^ Recently, certain new immunomodulatory properties of hCG have been identified to be critical for maternal tolerance of the embryo.^17^, ^18^ For example, hCG could suppress HLA‐DR expression but upregulate CD40 and CD80 expressions in innate lymphoid cells.^19^ hCG is capable of attracting Treg cells to the fetal–maternal interface, contacting maternal immune cells and orchestrating immune tolerance.^20^ hCG also enhances the suppressive activity of Treg cells.^21^ hCG can react with uterine natural killer cells (uNK) via the mannose receptor (MR, CD206) rather than the classical hCG/LH receptor, promoting the proliferation of uNK cells.^22^ hCG is also reported to reduce the ability of stimulated peripheral dendritic cells to secrete IL‐8 and IL‐10.^23^ However, the effects of hCG on peripheral γδT cells and the underlying mechanisms have not been described.

In this study, we conducted observations on the characteristics of the peripheral γδT cells obtained from women in early pregnancy and found that they exhibited higher expressions of CD69 and IL‐10 than healthy nonpregnant women. To investigate the potential roles of the increased hCG level in the altered immune functions of peripheral γδT cells, we performed cocultures of the purified peripheral γδT cells and the peripheral blood mononuclear cells (PBMCs) obtained from nonpregnant women, using hCG concentrations relevant to early pregnancy. Subsequently, we examined the effects of these cocultures on the proliferation, activation, and cytokine secretion of γδT cells. We hypothesized that there may be an indirect regulatory relationship between hCG and peripheral γδT cells, which plays a critical role in the establishment of immune tolerance during early pregnancy.

## 2 METHODS

### 2.1 Reagents

Commercial hCG was obtained from Livzon Company. The concentration of hCG was chosen according to early pregnancy‐relevant hCG levels found in normal pregnant women during the first trimester.

### 2.2 Blood samples

Fresh peripheral blood samples were collected from 12 healthy nonpregnant women who were counseling related to pregnancy and laboratory examinations and 15 pregnant women in early pregnancy (7–10 weeks of gestation) who received the routine physical examination at the obstetrics and gynecology department of the West China Second Hospital, between March 2022 and May 2022. The inclusion criteria involved the selection of women volunteers who do not have a history of serious illness and were above the age of 18. The exclusion criteria for women volunteers included autoimmune disorders, cardiovascular ailments, infection diseases, and pregnancy complications. We also recruited 5 healthy nonpregnant female community volunteers for the γδT cell isolation (30 mL of peripheral blood) and 12 healthy nonpregnant female community volunteers for the PBMC isolation (10 mL of peripheral blood). The protocol of specimen collection was approved by the Medical Ethics Committee of West China Second Hospital of Sichuan University (Record Number: 2020 (029)). The signed informed consent was obtained from all participants.

### 2.3 Purified γδT cell preparation

Peripheral γδT cells were purified by negative selection using immunomagnetic microbeads (Miltenyi Biotec, 130‐092‐892). The non‐γδT cells were indirectly magnetically labeled with a cocktail of biotinylated monoclonal antibodies and Anti‐Biotin MicroBeads. The magnetically labeled non‐TCRγδ^+^ cells were retained in a MACS® Column in the magnetic field of a MACS Separator, while the unlabeled γδT cells passed through the column. The purity of the isolated γδT cells was greater than 95%.

### 2.4 PBMC isolation

For PBMC isolation, the whole blood was diluted with an equal volume PBS before performing the centrifugation steps. Then, the blood was carefully added to the Ficoll (Tbdscience) layer (blood:Ficoll = 1:1) and centrifuged at 550*g* for 20 min with the brake off. The cellular layer at the Ficoll/PBS interface was aspirated and washed twice with PBS, removing the residual Ficoll. Approximately 1 × 10^6^ of PBMCs could be isolated from 1 mL of peripheral blood.

### 2.5 Cell culture

Purified peripheral γδT cells or isolated PBMCs were suspended in Roswell Park Memorial Institute 1640 medium (HyClone) supplemented with 10% fetal calf serum (Biological Industries) and 500 IU/mL recombinant human interleukin‐2 (rhIL‐2) (Sigma‐Aldrich). Approximately 1 × 10^5^ cells/well purified γδT cells and 5 × 10^5^ cells/well PBMCs were cultured in 24‐well flat‐bottom plates (Corning) in the absence or presence of early pregnancy‐relevant hCG levels (0, 25, 50, 100, and 200 IU/mL) at 37°C in a humidified atmosphere of 5% CO_2_ for 48 h. After 48 h of coculturing, the spent medium was removed, and the cells were washed with cold 1× PBS once for further flow cytometric analyses.

### 2.6 Flow cytometry

For cell surface markers, PBMCs and purified γδT cells were stained with anti‐human CD45, CD3, γδTCR, CD69, and NKG2D antibodies for 20 min in the dark.

For cytokine stimulation, the PMA/ionomycin/brefeldin A cocktail (catalog: 423303, BioLegend) was added to the cell suspension before tumor necrosis factor‐α (TNF‐α), interveron gamma (IFN‐γ), and IL‐17A staining, and the treated cells were incubated for 6 h at 37°C. The 100 ng/mL LPS (catalog: #14011, Cell Signaling Technology) was used to stimulate cells in the presence of monensin for 18 h at 37°C before IL‐10 staining.

For intracellular staining, the cells were fixed with fixation/permeabilization buffer (Invitrogen) for 1 h and stained with intracellular directly conjugated antibodies (CD206, Ki‐67, TNF‐α, IFN‐γ, IL‐17A, and IL‐10) for 20 min. Flow cytometry data were acquired on a FACS Celesta flow cytometer (BD), and analyzed using FlowJo software V.10. For the use of monoclonal antibodies, we referred to our published study^24^ and the details can be found in Supporting Information: Table 1.

### 2.7 PCR assay

The total RNA obtained from the purified peripheral γδT cells was converted to complementary DNA (cDNA) using an Evo M‐MLV RT kit with gDNA cleaned for qPCR II (AG111728, Accurate Biotechnology). PCR experiments were performed in a PCR thermal cycler (Bio‐Rad). GAPDH served as the internal control. Primer sequences are listed in Supporting Information: Table 2.

### 2.8 Immunofluorescence

Immunofluorescence assays of the purified peripheral γδT cells were carried out by Biossci Company. Blocking was performed in Tris‐buffered saline (Beyotime) with 10% normal serum (Biological Industries) and 0.1% Triton X‐100 (Beyotime). The 1:50 diluted rabbit anti‐human CD206 antibody (Abcam) and the 1:400 diluted donkey anti‐rabbit secondary antibody (Life Technologies) were used for the immunofluorescence assay. DAPI dye (Gibco) was used at room temperature for 20 min at a 1:500 dilution to identify the nucleus. The images were aquired on an Olympus BX53 microscope.

### 2.9 Statistical analysis

A Kolmogorov–Smirnov test was used to test for Gaussian distribution, followed by parametric or non‐parametric tests. The parametric test (unpaired *t*‐test) was utilized to compare two groups with normal distributions. The Mann‒Whitney *U* test was employed to compare two groups with non‐parametric distributions. The control group (untreated) and each of the hCG‐exposed groups were compared using ANOVA with Dunnett's multiple comparison test. Differences were considered significant when the *p* value < .05.

## 3 RESULTS

### 3.1 The percentages of peripheral CD69^+^ and IL‐10^+^ γδT cells were increased during early pregnancy

**Figure 1:**
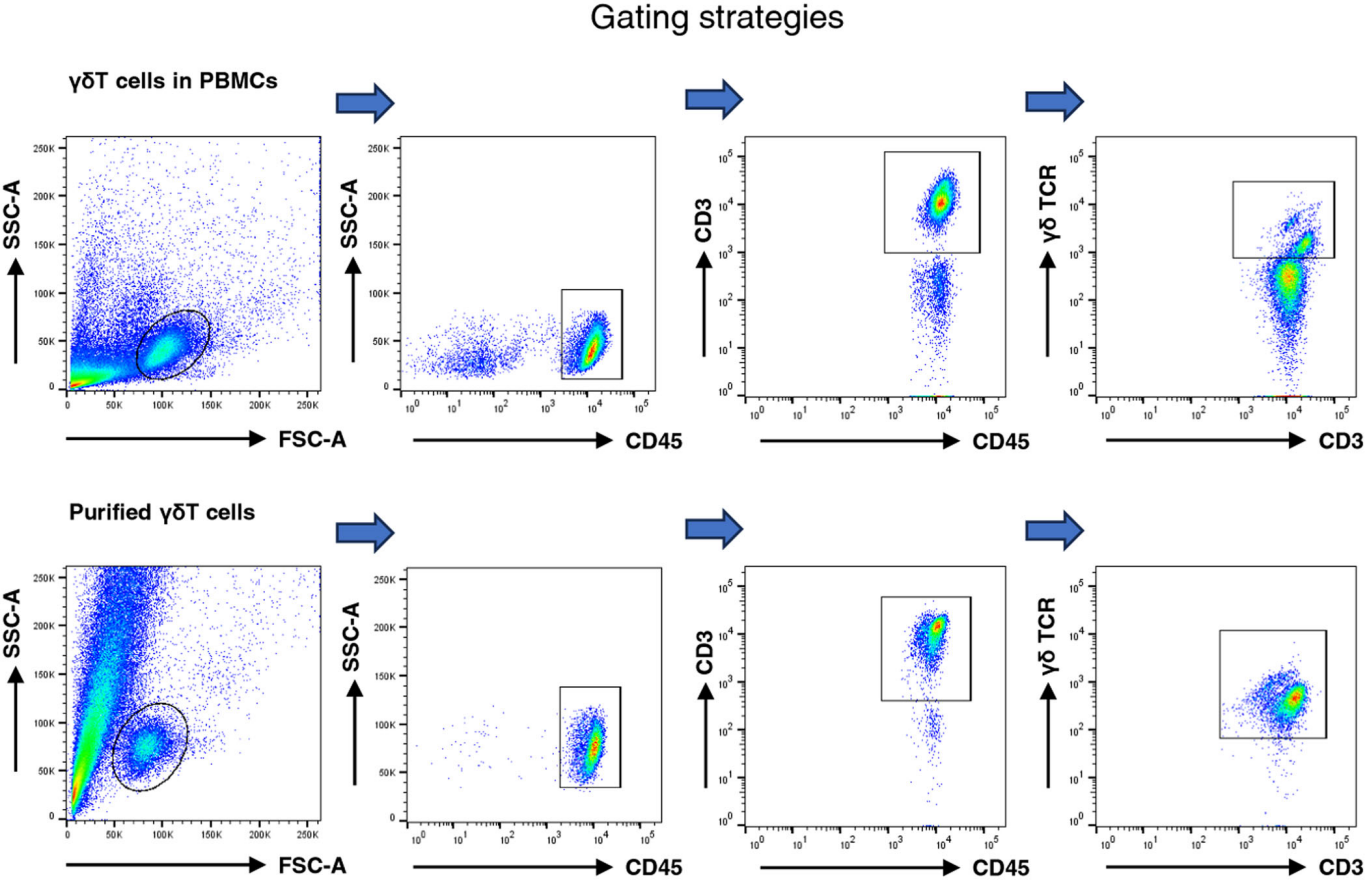
Gating strategy for γδT cells.

**Figure 2:**
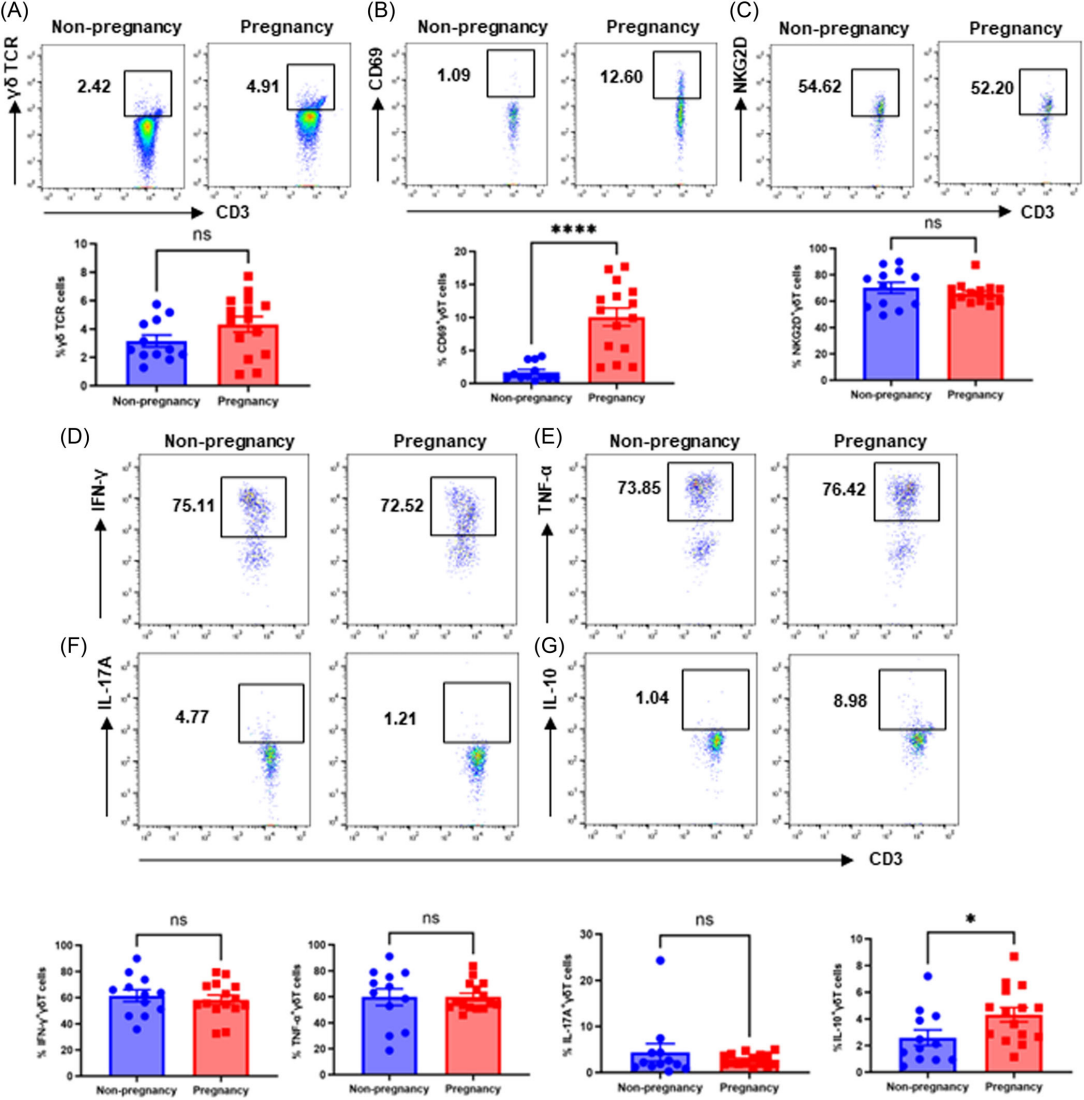
The changes in the characteristics of peripheral γδT cells in early pregnancy. (A) The ratios of peripheral γδT cells in the CD3^+^T cell population in nonpregnant and early pregnant groups were determined by flow cytometry assays. (B, C) The frequencies of peripheral CD69^+^γδT cells and NKG2D^+^γδT cells in nonpregnant and early pregnant groups were determined by flow cytometry assays. (D–G) The cytokine productions of peripheral γδT cells in nonpregnant and early pregnant groups were determined by flow cytometry assays. *N* = 12 for nonpregnant women and *N* = 15 for pregnant women in early pregnancy. ****p < .0001 and *p < .05. Error bars indicate mean ± SEM. A Kolmogorov–Smirnov test was used to test for Gaussian distribution, followed by parametric or non‐parametric tests. Two‐tailed *p* values were determined using Student's *t*‐test (ratios of peripheral NKG2D^+^γδT cells, IFN‐γ^+^γδT cells, and IL‐10^+^γδT cells) or Mann–Whitney test (ratios of peripheral γδT cells, CD69^+^γδT cells, TNF‐α^+^γδT cells, and IL‐17A^+^γδT cells).

To investigate the potential changes in the immunomodulatory functions of peripheral γδT cells during early pregnancy, we compared the proportion, activation, and cytokine production of γδT cells between healthy nonpregnant (median age, 26.8 years; range, 22–32 years; *N* = 12) women and early pregnant women (median age, 28.1 years; range, 22–34 years; gestational age, 7–10 weeks; *N* = 15). Gating strategies based on the published literature for the γδT cell were shown in Figure 1 and Supporting Information: Figure 1.^25^ Despite the absence of a statistically significant distinction in the proportion of γδT cells between the nonpregnant and early pregnant cohorts, the early pregnant individuals exhibited a tendency towards a higher frequency of γδT cells (4.33% ± 0.55%) compared to the nonpregnant individuals (3.15% ± 0.42%) (Figure 2A). Moreover, the peripheral γδT cells of early pregnant women showed a significantly elevated level of the early activation marker, CD69 (nonpregnancy: 1.73% ± 0.38% vs. early pregnancy: 10.06% ± 1.37%). There was no significant difference observed in the expression level of NKG2D, a surface receptor crucial for the activation of NK cells, CD8^+^T cells, and γδT cells,^26^ between the two groups (nonpregnancy: 70.05% ± 4.20% vs. early pregnancy: 65.64% ± 2.01% in) (Figure 2B,C). Compared with nonpregnant women, women in early pregnancy showed an enhanced ratio of IL‐10^+^γδT cells, which may contribute to the maintenance of immune tolerance (non‐pregnancy: 2.59% ± 0.58% vs. early pregnancy: 4.31% ± 0.54%). However, there were no significant differences in the expressions of certain pro‐inflammatory cytokines between the two groups, including TNF‐α (nonpregnancy: 59.73% ± 6.47% vs. early pregnancy: 59.80% ± 2.86%), IFN‐γ (nonpregnancy: 61.49% ± 4.47% vs. early pregnancy: 58.34% ± 3.54%), and IL‐17A (nonpregnancy: 4.37% ± 1.90% vs. early pregnancy: 2.80% ± 0.33%) (Figure 2D–G and Supporting Information: Figure 2). These results demonstrate that γδT cells are activated and secrete more IL‐10 during early pregnancy, playing roles in the induction of immunological tolerance.

### 3.2 The potential absence of substantial direct impacts of hCG on purified peripheral γδT cells

**Figure 3:**
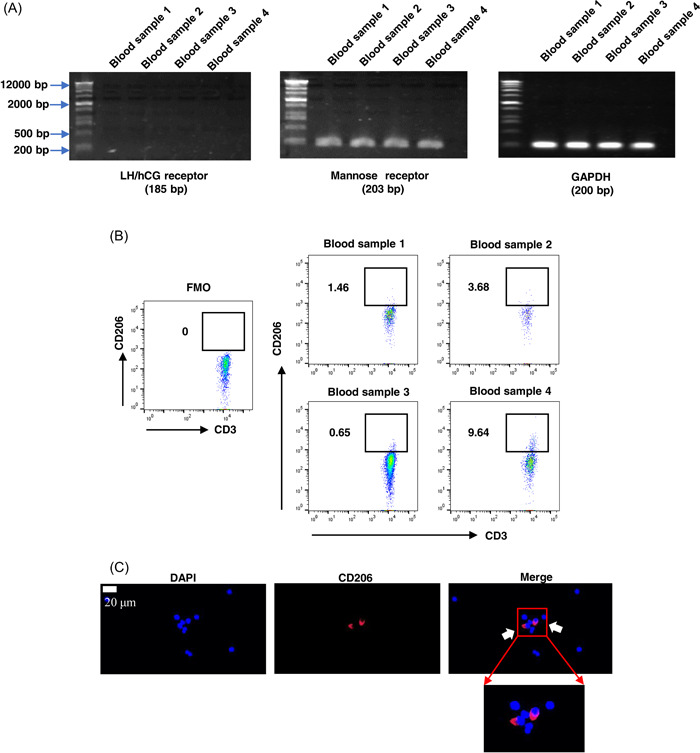
The expression of the mannose receptor (CD206) for human chorionic gonadotrophin (hCG) in peripheral γδT cells. (A) The mannose receptor mRNA expressions were detected in all samples of purified peripheral γδT cells while these samples did not show measurable luteinizing hormone (LH)/hCG receptor mRNA expressions via PCR and agarose gel electrophoresis assays (*N* = 4). (B) Flow cytometry analysis for the mannose receptor (CD206) expression in the peripheral γδT cells (*N* = 4). (C) Immunofluorescence staining of the purified peripheral γδT cells with CD206. Scale bar, 20 μm.

**Figure 4:**
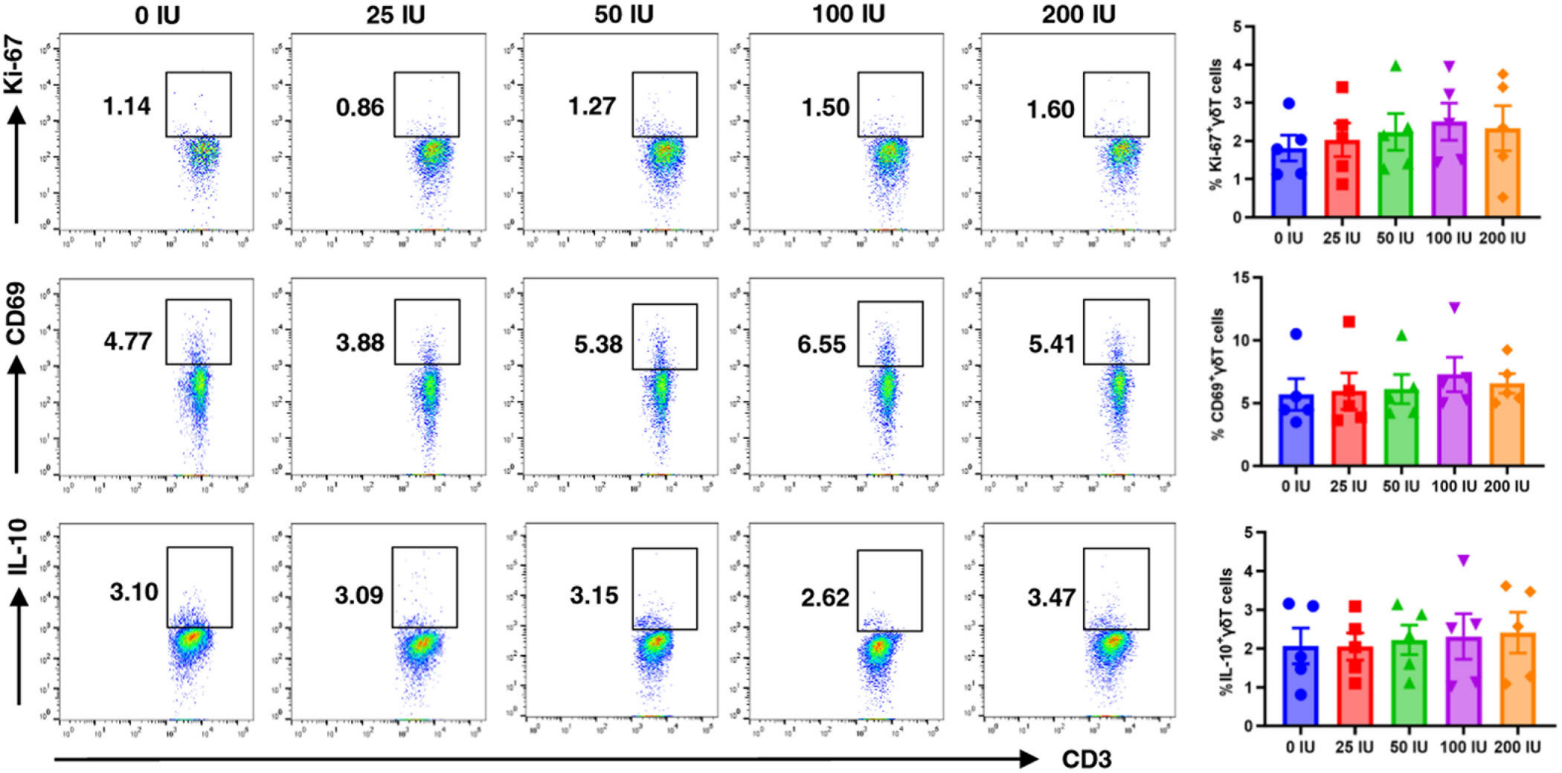
Human chorionic gonadotrophin (hCG) may have no significant direct effects on the purified peripheral γδT cells. The administration of early pregnant‐revenant hCG concentrations directly to purified peripheral γδT cells, may not yield significant alterations in the expressions of Ki‐67, CD69, and IL‐10 (%) within the purified γδT cells. *N* = 5. Error bars indicate mean ± SEM. Control group (untreated) and each of the hCG‐exposed groups were compared using ANOVA with Dunnett's multiple comparison test.

To explore whether above changes in immune functions of peripheral γδT cells are associated with the increasing release of hCG in the peripheral circulation during early pregnancy, we assessed the expressions of receptors for hCG (LH/hCG or mannose) in γδT cells. We purified γδT cells from the PBMCs of four nonpregnant women via magnetic bead sorting. Then, the RNA was extracted from these highly purified γδT cells and PCR experiments were applied using primers specific for LH/hCG receptor and mannose receptor (MR) mRNA. The results showed that MR mRNA was detected in all samples of purified peripheral γδT cells while these samples did not exhibit measurable LH/hCG receptor expression (Figure 3A). We also examined the expression of MR protein (CD206) in γδT cells using a flow cytometry assay with a specific anti‐CD206 antibody. According to the previous method for the measurement of CD206 in uNK cells, cells were fixed and permeabilized before immunostaining for CD206 since it is internalized following binding to carbohydrates.^22^ The results showed that only approximately 1%–10% of peripheral γδT cells stained positive for CD206 (Figure 3B). Further immunofluorescence assay also suggested that a fraction of purified γδT cells significantly expressed mannose receptors (Figure 3C). Next, the purified γδT cells (1 × 10^5^ cells/mL) obtained from nonpregnant women (*N* = 5) were cultured in the absence or presence of early pregnancy‐relevant hCG concentrations (0, 25, 50, 100, and 200 IU/mL) at 37°C for 48 h. However, we found that the direct treatment of purified γδT cells with above hCG concentrations may have no significant effects on their Ki‐67, CD69, and IL‐10 expression levels (%) (Figure 4). These results demonstrate that hCG do not exert a direct effect on the Ki‐67, CD69, and IL‐10 expressions in peripheral γδT cells under the experimental conditions we used, which might be attributed to the relatively limited expressions of mannose receptors (CD206) in γδT cells.

### 3.3 The impact of hCG on peripheral γδT cells is mediated through the regulation of the immune microenvironment

**Figure 5:**
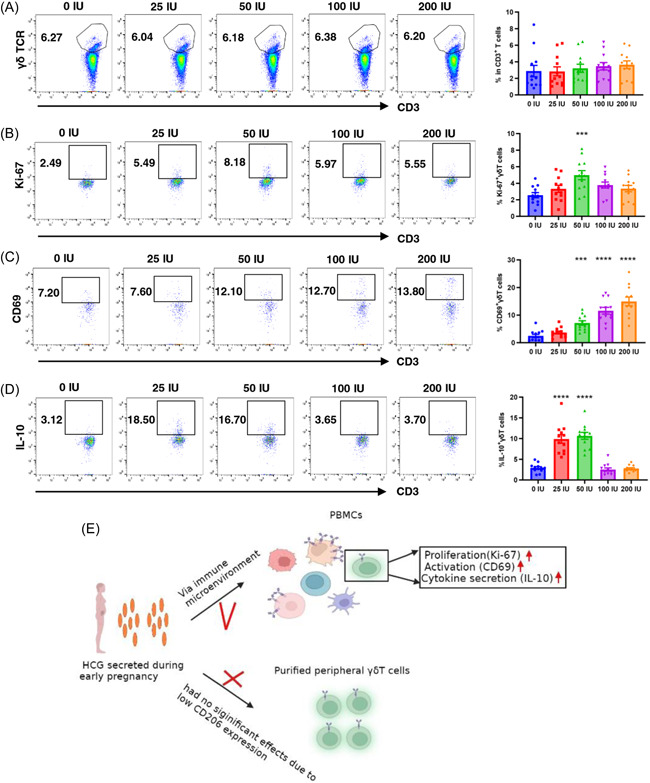
Human chorionic gonadotrophin (hCG) indirectly enhances the ratios of peripheral Ki‐67^+^γδT cells, CD69^+^γδT cells, and IL‐10^+^γδT cells in PBMCs. (A) When peripheral blood mononuclear cells (PBMCs) were cocultured with the early pregnancy‐relevant hCG concentrations, the percentages of peripheral γδT cells in the CD3^+^T cell population were not significantly changed via flow cytometry assays. (B) The ratio of peripheral Ki‐67^+^γδT cells in total γδT cells was only elevated at a 50 IU/mL concentration of hCG. (C) The ratios of peripheral CD69^+^γδT cells were increased and appeared to be in a hCG dose‐dependent manner. (D) At 25 and 50 IU/mL concentration of hCG, increased ratios of peripheral IL‐10^+^γδT cells were observed. (E) A schematic illustration of hCG could indirectly mediate the immune functions of γδT cells, which may depend on the changed immune microenvironment. ***p < .001, ****p < .0001. *N* = 12. Error bars indicate mean ± SEM. Control group (untreated) and each of the hCG‐exposed groups were compared using ANOVA with Dunnett's multiple comparison test.

Although we suggested the lack of direct effects of hCG on peripheral γδT cells, whether hCG could indirectly impact γδT cells via the immune microenvironment is worth further exploration. To address this question, PBMCs (5 × 10^5^ cells/mL) obtained from nonpregnant women were cultured in 24‐well flat‐bottom plates in the absence or presence of the same concentrations of hCG (0, 25, 50, 100, and 200 IU/mL) at 37°C for 48 h. We evaluated the percentage of peripheral γδT cells in the CD3^+^T cell population and ratios of Ki‐67^+^γδT cells, CD69^+^γδT cells, and IL‐10^+^γδT cells in the total γδT cell population. After 48 h of coculture, the proportions of γδT cells in CD3^+^T cells were still not significantly altered (Figure 5A). However, the addition of 50 IU/mL hCG may result in a significant increase in the proliferation potential of γδT cells based on the enhanced expression of Ki‐67, whereas higher doses (100 and 200 IU/mL) did not seem to have similar obvious effects (Figure 5B). In addition, hCG treatment led to an increase in the frequency of CD69^+^γδT cells in a concentration‐dependent manner (Figure 5C). Significant increases were also observed in IL‐10^+^γδT cells in response to the treatments with 25 and 50 IU/mL hCG (Figure 5D). These data suggest that increased hCG concentration during early pregnancy may indirectly cause the activation, proliferation, and increased IL‐10 secretion of peripheral γδT cells by changing immune microenvironment (Figure 5E).

## 4 DISCUSSION

γδT cells were shown to be able to secrete a variety of anti‐inflammatory cytokines, including TGF‐β, IL‐10, and IL‐4, in early pregnancy, facilitating the establishment of an immunotolerance microenvironment.^5^, ^8^, ^27^ hCG is one of the earliest proteins secreted by fetal trophoblasts and the importance of hCG in pregnancy is well established.^28^, ^29^ In this study, we have provided evidence indicating that hCG has the potential to indirectly enhance the expression levels of CD69 and IL‐10 in peripheral γδT cells across various concentrations. This phenomenon may contribute to the establishment of an immunologically tolerant environment.

It has been previously shown that certain functions and phenotypes of immune cells, such as NK cells and Treg cells, were altered in early pregnancy.^30^, ^31^, ^32^ In this study, we observed peripheral γδT cells underwent dynamic changes in immune functional characteristics in early pregnancy, such as the increased CD69 and IL‐10 expressions, which were consistent with the data of Olga Nagaeva et al.^6^ Previous studies have proven that hCG can act on its receptors and activate intracellular pathways.^33^ Thus, we proceeded to investigate the impact of increased serum hCG levels during early pregnancy on the immune function of peripheral γδT cells. Specifically, we assessed the expression levels of hCG receptors, namely the LH/hCG receptor and mannose receptor (CD206), in γδT cells. The expression of mannose receptor was observed in purified γδT cells, whereas the expression of LH/hCG receptor was not detected. This finding closely resembled the expression pattern for hCG receptor in uterine NK cells.^22^ Next, an unforeseen outcome emerged, as the administration of hCG did not yield any noteworthy impacts on the purified γδT cells obtained from nonpregnant women using the coculture technique outlined in our study. We hypothesized that this phenomenon may be attributed to the limited expressions of mannose receptors in γδT cells, resulting in the feeble interactions. Notably, studies performed on cell culture models may be influenced by culture conditions.^34^ It was important to acknowledge that our purified γδT cells were subjected to a specific coculture condition (1640RPIM + 10% FBS + 500 IU/mL IL‐2 + hCG). It remained uncertain whether altering the culture conditions, such as pre‐stimulating the cells with anti‐CD3/CD28 or extending the co‐culture period, could potentially influence the outcomes.

The role of the immune microenvironment in the control of functions of immune cells has been demonstrated, suggesting that the outcome of the immune response is not completely determined by antigen‐immune cell interactions.^35^, ^36^ Thus, we cocultured PBMCs isolated from nonpregnant women with early pregnancy revenant hCG concentrations. Interestingly, in the context of the immune microenvironment, despite a nonsignificant alteration in the total peripheral γδT cell percentage was still observed, the percentage of peripheral Ki‐67^+^γδT cells was increased at a concentration of 50 IU/mL hCG. The ratio of peripheral CD69^+^γδT cells was enhanced and appeared to be dose dependent. Significant increases in IL‐10 productions in γδT cells were also observed at concentrations of 25 and 50 IU/mL hCG. Nan Yu et al. have proposed that hCG could promote trophoblast invasion by stimulating the cytokine secretion of human PBMCs.^37^ Overall, we hypothesized that hCG may induce the proliferation and activation of peripheral γδT cells by altering the immune microenvironment during early pregnancy.

There are still several limitations of our study. First, we only explored the effects of hCG on peripheral γδT cells, and our results may not represent the situation at the maternal–fetal interface due to the obvious differences between peripheral γδT cells and tissue resident γδT cells.^38^ It is also worth noting that an upsurge occurs in the serum hCG soon after fertilization, peaks at 8–10 gestational weeks, and then continues to decline.^39^ Such a dynamic change in the serum hCG concentration may have different effects on the immune function of peripheral γδT cells. The effects of different pregnancy‐related hCG concentrations at the different points of pregnancy (such as <6 weeks and 10–12 weeks gestation) merit exploration. In addition, the hCG values of patients with miscarriage usually decrease,^40^ and the comparisons between normal nonpregnant women, normal pregnant women, and miscarriage women (related hCG concentrations) are also worth studying in the future. We only proposed certain immune effective functions of peripheral γδT cells may be indirectly affected by hCG; however, it has been reported that γδT cells are capable of secreting growth factors in early pregnancy, such as growth differentiation factor 15 (GDF15) and bone morphogenetic protein 1 (BMP1).^24^ Whether hCG can affect more versatile functions of γδT cells is also worth investigating.

## 5 CONCLUSIONS

Peripheral γδT cells contribute to establishing immune tolerance via increased IL‐10 production in early pregnancy. Increased hCG concentration in the blood circulation may not directly affect certain functions of γδT cells because of the low expressions of mannose receptors in γδT cells. However, the addition of pregnancy‐relevant concentrations of hCG to PBMCs can indirectly activate peripheral γδT cells to produce more IL‐10, which may be dependent on immune microenvironment modulation (Figure 5E).

## AUTHOR CONTRIBUTIONS

Liman Li performed the experiments and drafted the manuscript. Wenjie Zhou collected clinical samples. Yuan Liu performed PCR assays and cell culture assays. Chuan Yang and Ting Feng performed flow cytometry assays. Hong Li was liable for oversight and leadership responsibility for research activity planning and execution.

## CONFLICT OF INTEREST STATEMENT

The authors declare no conflict of interest.

## Supporting information

Supporting information.

## Data Availability

All data used to support the findings of this study are available from the corresponding author upon reasonable request.

## References

[1] Alexandrova M , Manchorova D , Dimova T . Immunity at maternal‐fetal interface: KIR/HLA (Allo)recognition. Immunol Rev. 2022;308(1):55‐76. 35610960 10.1111/imr.13087

[2] Qin D , Xu H , Chen Z , et al. The peripheral and decidual immune cell profiles in women with recurrent pregnancy loss. Front Immunol. 2022;13:994240. 36177021 10.3389/fimmu.2022.994240 PMC9513186

[3] Cai D , Tang Y , Yao X . Changes of γδT cell subtypes during pregnancy and their influences in spontaneous abortion. J Reprod Immunol. 2019;131:57‐62. 30710888 10.1016/j.jri.2019.01.003

[4] Mincheva‐Nilsson L , Hammarström S , Hammarström ML . Human decidual leukocytes from early pregnancy contain high numbers of gamma delta+ cells and show selective down‐regulation of alloreactivity. J Immunol. 1992;149(6):2203‐2211. 1381400

[5] Xu QH , Liu H , Wang LL , et al. Roles of gammadeltaT cells in pregnancy and pregnancy‐related complications. Am J Reprod Immunol. 2021;86(5):e13487. 34331364 10.1111/aji.13487

[6] Nagaeva O , Jonsson L , Mincheva‐Nilsson L . Dominant IL‐10 and TGF‐β mRNA expression in γδT cells of human early pregnancy decidua suggests immunoregulatory potential. Am J Reprod Immunol. 2002;48(1):9‐17. 12322898 10.1034/j.1600-0897.2002.01131.x

[7] Clark DA , Croitoru K . TH1/TH2,3 imbalance due to cytokine‐producing NK, γδ T and NK‐γδ T cells in murine pregnancy decidua in success or failure of pregnancy. Am J Reprod Immunol. 2001;45(5):257‐265. 11432400 10.1111/j.8755-8920.2001.450501.x

[8] Huang C , Zeng Y , Tu W . The role of gammadelta‐t cells during human pregnancy. Am J Reprod Immunol. 2017;78(2):e12713. 10.1111/aji.12713 28653491

[9] Mincheva‐Nilsson L , Nagaeva O , Sundqvist KG , Hammarström ML , Hammarström S , Baranov V . γδ T cells of human early pregnancy decidua: evidence for cytotoxic potency. Int Immunol. 2000;12(5):585‐596. 10784604 10.1093/intimm/12.5.585

[10] Guo R , Jiang S , Zhang J , et al. PD‐1 mediates decidual gammadelta T cells cytotoxicity during recurrent pregnancy loss. Am J Reprod Immunol. 2022;88(3):e13562. 35567369 10.1111/aji.13562

[11] Ma L , Papadopoulou M , Taton M , et al. Effector Vγ9Vδ2 T cell response to congenital *Toxoplasma gondii* infection. JCI Insight. 2021;6(16):e138066. 34255746 10.1172/jci.insight.138066 PMC8409983

[12] Miller D , Motomura K , Galaz J , et al. Cellular immune responses in the pathophysiology of preeclampsia. J Leukoc Biol. 2022;111(1):237‐260. 33847419 10.1002/JLB.5RU1120-787RR PMC8511357

[13] Lazzaretti C , Secco V , Paradiso E , et al. Identification of key receptor residues discriminating human chorionic gonadotropin (hCG)‐ and luteinizing hormone (LH)‐specific signaling. Int J Mol Sci. 2020;22(1):151. 33375708 10.3390/ijms22010151 PMC7794846

[14] d'Hauterive SP , Close R , Gridelet V , Mawet M , Nisolle M , Geenen V . Human chorionic gonadotropin and early embryogenesis: review. Int J Mol Sci. 2022;23(3):1380. 35163303 10.3390/ijms23031380 PMC8835849

[15] Jaffe RB , Lee PA , Midgley Jr., AR . Serum gonadotropins before, at the inception of, and following human pregnancy. J Clin Endocrinol Metab. 1969;29(9):1281‐1283. 5808533 10.1210/jcem-29-9-1281

[16] Seeber BE . What serial hCG can tell you, and cannot tell you, about an early pregnancy. Fertil Steril. 2012;98(5):1074‐1077. 23031160 10.1016/j.fertnstert.2012.09.014

[17] Fang L , Wang S , Han X , et al. Amphiregulin stimulates human chorionic gonadotropin expression by inducing ERK1/2‐mediated ID3 expression in trophoblast cells. Placenta. 2021;112:73‐80. 34329970 10.1016/j.placenta.2021.07.292

[18] Borisova MA , Moiseenko DY , Smirnova OV . Human chorionic gonadotropin: unknown about known. Hum Physiol. 2017;43(1):93‐104. 29509368

[19] Einenkel R , Ehrhardt J , Hartmann K , Krüger D , Muzzio DO , Zygmunt M . Hormonally controlled ILC antigen presentation potential is reduced during pregnancy. Reproduction. 2020;160(1):155‐169. 32130203 10.1530/REP-19-0554

[20] Schumacher A , Brachwitz N , Sohr S , et al. Human chorionic gonadotropin attracts regulatory T cells into the fetal‐maternal interface during early human pregnancy. J Immunol. 2009;182(9):5488‐5497. 19380797 10.4049/jimmunol.0803177

[21] Schumacher A , Heinze K , Witte J , et al. Human chorionic gonadotropin as a central regulator of pregnancy immune tolerance. J Immunol. 2013;190(6):2650‐2658. 23396945 10.4049/jimmunol.1202698

[22] Kane N , Kelly R , Saunders PTK , Critchley HOD . Proliferation of uterine natural killer cells is induced by human chorionic gonadotropin and mediated via the mannose receptor. Endocrinology. 2009;150(6):2882‐2888. 19196802 10.1210/en.2008-1309 PMC2709965

[23] Sauss K , Ehrentraut S , Zenclussen AC , Schumacher A . The pregnancy hormone human chorionic gonadotropin differentially regulates plasmacytoid and myeloid blood dendritic cell subsets. Am J Reprod Immunol. 2018;79(4):e12837. 29488661 10.1111/aji.12837

[24] Yang S , Feng T , Ma C , et al. Early pregnancy human decidua gamma/delta T cells exhibit tissue resident and specific functional characteristics. Mol Hum Reprod. 2022;28(8): gaac023. 35758607 10.1093/molehr/gaac023

[25] Barros‐Martins J , Bruni E , Fichtner AS , Cornberg M , Prinz I . OMIP‐084: 28‐color full spectrum flow cytometry panel for the comprehensive analysis of human gammadelta T cells. Cytometry A. 2022;101(10):856‐861. 35521651 10.1002/cyto.a.24564

[26] Lerner EC , Woroniecka KI , D'Anniballe VM , et al. CD8(+) T cells maintain killing of MHC‐I‐negative tumor cells through the NKG2D‐NKG2DL axis. Nat Cancer. 2023;4(9):1258‐1272. 37537301 10.1038/s43018-023-00600-4 PMC10518253

[27] Liu H , Lin XX , Huang XB , et al. Systemic characterization of novel immune cell phenotypes in recurrent pregnancy loss. Front Immunol. 2021;12:657552. 34122414 10.3389/fimmu.2021.657552 PMC8195235

[28] Gridelet V , Perrier d'Hauterive S , Polese B , Foidart JM , Nisolle M , Geenen V . Human chorionic gonadotrophin: new pleiotropic functions for an “old” hormone during pregnancy. Front Immunol. 2020;11:343. 32231662 10.3389/fimmu.2020.00343 PMC7083149

[29] Schumacher A , Zenclussen AC . Human chorionic gonadotropin‐mediated immune responses that facilitate embryo implantation and placentation. Front Immunol. 2019;10:2896. 31921157 10.3389/fimmu.2019.02896 PMC6914810

[30] Bulmer JN , Lash GE . The role of uterine NK cells in normal reproduction and reproductive disorders. Adv Exp Med Biol. 2015;868:95‐126. 26178847 10.1007/978-3-319-18881-2_5

[31] Saito S . Reconsideration of the role of regulatory T cells during pregnancy: differential characteristics of regulatory T cells between the maternal‐fetal interface and peripheral sites and between early and late pregnancy. Med Princ Pract. 2022;31(5):403‐414. 36195068 10.1159/000527336 PMC9801372

[32] Leber A , Teles A , Zenclussen AC . Regulatory T cells and their role in pregnancy. Am J Reprod Immunol. 2010;63(6):445‐459. 20331584 10.1111/j.1600-0897.2010.00821.x

[33] Stewart JL , Gao L , Flaws JA , et al. Effects of nerve growth factor‐beta from bull seminal plasma on steroidogenesis and angiogenic markers of the bovine pre‐ovulatory follicle wall cell culture. Front Vet Sci. 2021;8:786480. 35111838 10.3389/fvets.2021.786480 PMC8801700

[34] Rossmeislová L , Mališová L , Kračmerová J , et al. Weight loss improves the adipogenic capacity of human preadipocytes and modulates their secretory profile. Diabetes. 2013;62(6):1990‐1995. 23378611 10.2337/db12-0986 PMC3661637

[35] Rotta G , Matteoli G , Mazzini E , Nuciforo P , Colombo MP , Rescigno M . Contrasting roles of SPARC‐related granuloma in bacterial containment and in the induction of anti‐*Salmonella typhimurium* immunity. J Exp Med. 2008;205(3):657‐667. 18316416 10.1084/jem.20071734 PMC2275387

[36] Wang Y , Misumi I , Gu AD , et al. GATA‐3 controls the maintenance and proliferation of T cells downstream of TCR and cytokine signaling. Nat Immunol. 2013;14(7):714‐722. 23708251 10.1038/ni.2623 PMC3688666

[37] Yu N , Yan W , Yin T , et al. HCG‐activated human peripheral blood mononuclear cells (PBMC) promote trophoblast cell invasion. PLoS One. 2015;10(6):e0125589. 26087261 10.1371/journal.pone.0125589 PMC4472760

[38] Kang S , Wu Q , Huang J , et al. Tissue resident memory gammadeltat cells in murine uterus expressed high levels of IL‐17 promoting the invasion of trophocytes. Front Immunol. 2020;11:588227. 33519808 10.3389/fimmu.2020.588227 PMC7840782

[39] Friis Petersen J , Friis‐Hansen LJ , Jensen AK , Nyboe Andersen A , Løkkegaard ECL . Early pregnancy reference intervals; 29 serum analytes from 4 to 12 weeks’ gestation in naturally conceived and uncomplicated pregnancies resulting in live births. Clin Chem Lab Med. 2019;57(12):1956‐1967. 31343977 10.1515/cclm-2019-0495

[40] Liu Y , Lv W . The diagnostic value of transvaginal color Doppler ultrasonography plus serum β‐HCG dynamic monitoring in intrauterine residue after medical abortion. Medicine. 2023;102(5):e31217. 36749252 10.1097/MD.0000000000031217 PMC9901960

