# Supplementary material for: Human chorionic gonadotrophin indirectly activates peripheral γδT cells to produce interleukin‐10 during early pregnancy
Source: Immun Inflamm Dis. 2024 Jan 10;12(1):e1119. doi: 10.1002/iid3.1119 (PMC10777880; doi:10.1002/iid3.1119)
Supplement: Supplementary file 1 — Supporting information. [file IID3-12-e1119-s001.docx]

**SI Table 1. The list of antibodies for flow cytometry**

| Antibody | Dilution | Source and Identifier |
| --- | --- | --- |
| CD45-APC/CY7 | 1:100 | BioLegend (Catalog: 304014, Clone: HI3) |
| CD3- PerCP/Cy5.5 | 1:100 | BioLegend (Catalog: 300430, Clone: UCHT1) |
| CD3-FITC | 1:100 | BioLegend (Catalog: 300406, Clone: UCHT1) |
| TCR γδ-PE | 1:50 | BioLegend (Catalog: 331210, Clone: B1) |
| TCR γδ-APC | 1:50 | BioLegend (Catalog: 331212, Clone: B1) |
| CD206-PE | 1:50 | Proteintech (Catalog: PE65155, Lot: 51000355) |
| CD69-PerCP/Cy5.5 | 1:50 | BioLegend (Catalog: 310926, Clone: FN50) |
| NKG2D-PE | 1:50 | BioLegend (Catalog: 320806, Clone: 1D11) |
| IFN-γ-FITC | 1:50 | BioLegend (Catalog: 506504, Clone: B27) |
| TNF-α-APC | 1:50 | BioLegend (Catalog: 502912, Clone: MAb11) |
| IL-17A-BV605 | 1:50 | BioLegend (Catalog: 512326, Clone: BL168) |
| IL-10-BV421 | 1:50 | BioLegend (Catalog: 501422, Clone: JES3-9D7) |

**SI Table 2. The list of primer sequences**

| Genes | Forward | Reverse | |  |
| --- | --- | --- | --- | --- |
| *LH/hCG receptor* | 5’-ATGAAGCAGCGGTTCTCG -3’ | 5’-TTGACAGGGAGGTAGGCAAG-3’ |  |  |
| *Mannose receptor* | 5’-CTACCCCTGCTCCTGGTTTTT -3’ | 5’-TGAAACACTCATAATCTGAGATTC-3’ | | |
| *GAPDH* | 5’-ATGACATCAAGAAGGTGGTG-3’ | 5’-ATGACATCAAGAAGGTGGTG-3’ | | |

**SI Table 3. Details around the donor cohort**

|  | Maternal age (**year)** | BMI  (**kg/m^2^)** | Gestational age **(week)** | No. prior pregnancy |
| --- | --- | --- | --- | --- |
| nonpregnant woman 1 | 28 | 21.4 | / | 0 |
| nonpregnant woman 2 | 26 | 22.9 | / | 0 |
| nonpregnant woman 3 | 22 | 22.7 | / | 0 |
| nonpregnant woman 4 | 27 | 21.6 | / | 1 |
| nonpregnant woman 5 | 26 | 20.6 | / | 0 |
| nonpregnant woman 6 | 25 | 20.2 | / | 0 |
| nonpregnant woman 7 | 26 | 23.5 | / | 0 |
| nonpregnant woman 8 | 29 | 22.8 | / | 1 |
| nonpregnant woman 9 | 30 | 21.9 | / | 0 |
| nonpregnant woman 10 | 32 | 23.1 | / | 0 |
| nonpregnant woman 11 | 22 | 20.9 | / | 0 |
| nonpregnant woman 12 | 29 | 22.2 | / | 0 |
| pregnant woman 1 | 29 | 23.6 | 7 | 0 |
| pregnant woman 2 | 25 | 22.3 | 8 | 0 |
| pregnant woman 3 | 27 | 21.4 | 7 | 0 |
| pregnant woman 4 | 29 | 21.8 | 10 | 1 |
| pregnant woman 5 | 26 | 21 | 9 | 0 |
| pregnant woman 6 | 32 | 23.5 | 9 | 0 |
| pregnant woman 7 | 33 | 23 | 9 | 0 |
| pregnant woman 8 | 34 | 22.4 | 8 | 1 |
| pregnant woman 9 | 29 | 21.7 | 7 | 0 |
| pregnant woman 10 | 26 | 22.9 | 10 | 0 |
| pregnant woman 11 | 32 | 20.2 | 7 | 0 |
| pregnant woman 12 | 28 | 23.3 | 7 | 0 |
| pregnant woman 13 | 29 | 22.1 | 7 | 1 |
| pregnant woman 14 | 22 | 23.6 | 8 | 0 |
| pregnant woman 15 | 21 | 23.2 | 9 | 0 |


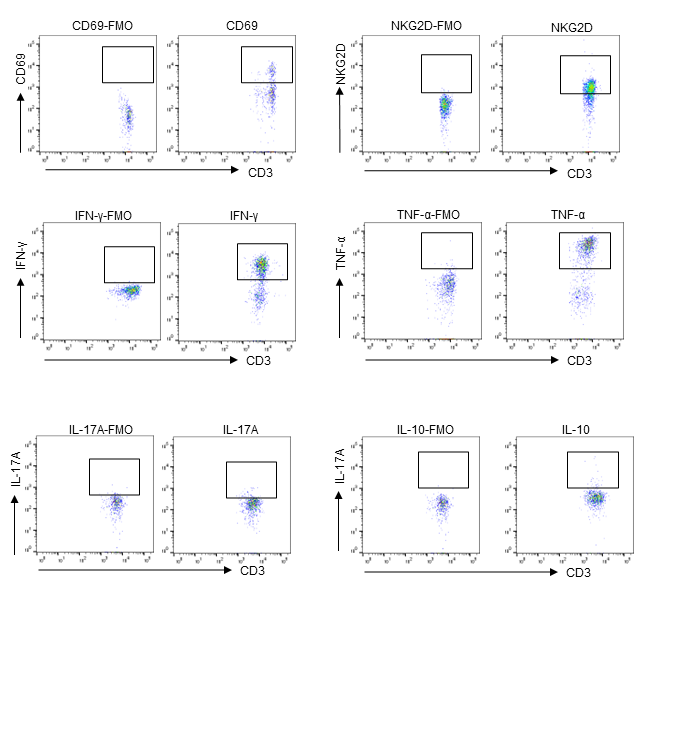


**SI Figure 1** FMO controls for cytokine/surface markers.


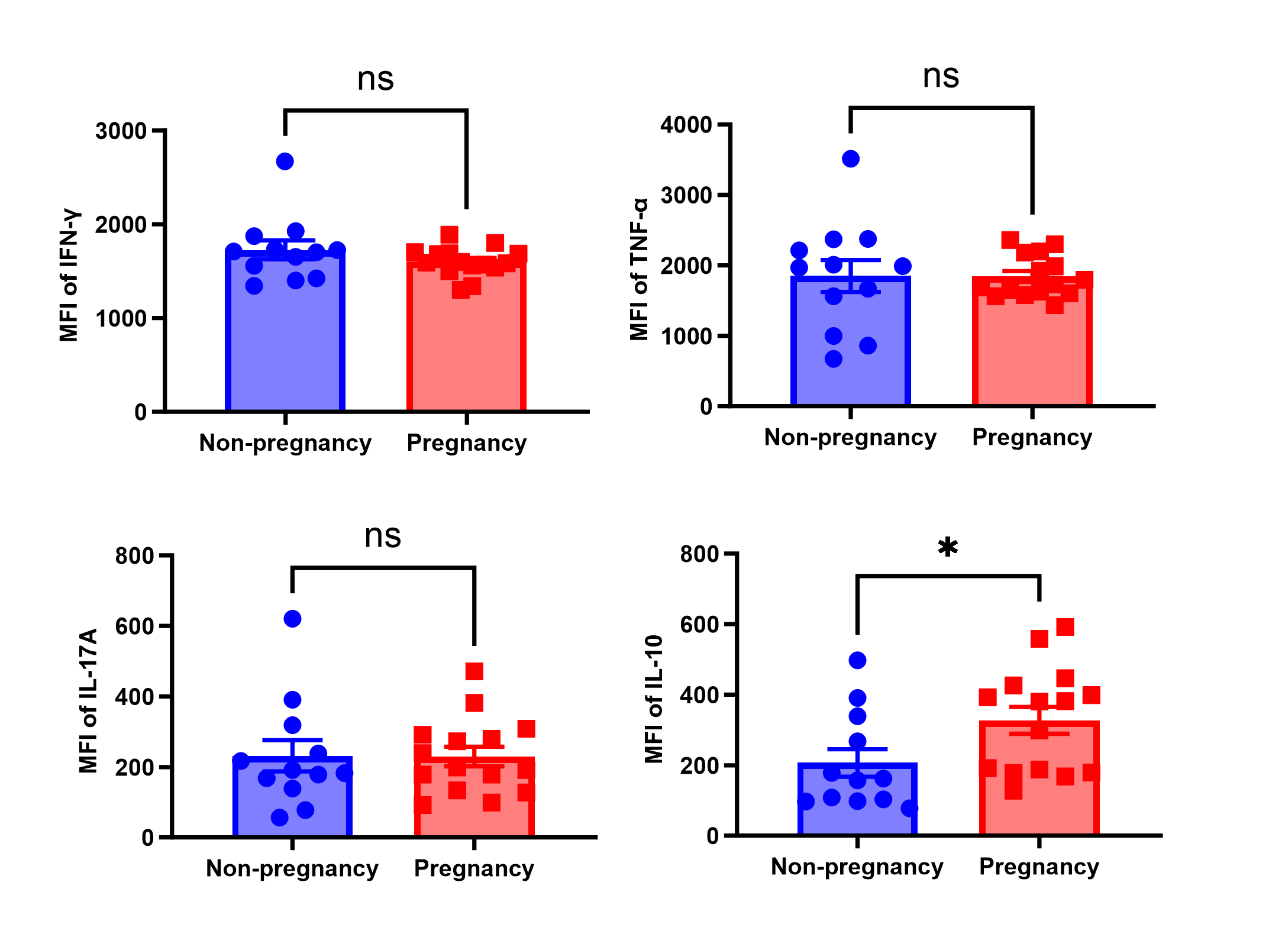


**SI Figure 2 The mean fluorescence intensity (MFI) values of cytokines.** Flow cytometry assays were employed to determine the MFI of IFN-γ, TNF-α, IL-17A, and IL-10 in peripheral γδT cells within both nonpregnant and early pregnant groups. The results revealed that the MFI of IL-10 exhibited statistical significance between the 2 groups, whereas no notable disparities were observed in the remaining pro-inflammatory factors. Error bars indicate mean ± SEM. A Kolmogorov–Smirnov test was used to test for Gaussian distribution, followed by parametric or non‐parametric tests. Two-tailed p-values were determined using Student’s t test (TNF-α and IL-17A) or Mann–Whitney test (IFN-γ and IL-10).
